# Antinociceptive effects of magnesium sulfate for monitored anesthesia care during hysteroscopy: a randomized controlled study

**DOI:** 10.1186/s12871-020-01158-9

**Published:** 2020-09-21

**Authors:** Peng-fei Gao, Jing-yan Lin, Shun Wang, Yun-feng Zhang, Guo-qiang Wang, Qi Xu, Xiao Guo

**Affiliations:** 1 Department of Anesthesiology, North Sichuan Medical College, Nanchong, 637000 Sichuan China; 2 Department of Anesthesiology, Affiliated Hospital of North Sichuan Medical College, Nanchong, 637000 Sichuan China

**Keywords:** Hysteroscopy, Magnesium sulphate, Monitored anesthesia care, Opioid, Pain

## Abstract

**Background:**

Opioids are the most effective antinociceptive agents, they have undesirable side effects such as respiratory depressant and postoperative nausea and vomiting. The purpose of the study was to evaluate the antinociceptive efficacy of adjuvant magnesium sulphate to reduce intraoperative and postoperative opioids requirements and their related side effects during hysteroscopy.

**Methods:**

Seventy patients scheduled for hysteroscopy were randomly divided into 2 groups. Patients in the magnesium group (Group M) received intravenous magnesium sulfate 50 mg/kg in 100 ml of isotonic saline over 15 min before anesthesia induction and then 15 mg/kg per hour by continuous intravenous infusion. Patients in the control group (Group C) received an equal volume of isotonic saline as placebo. All patients were anesthetized under a BIS guided monitored anesthesia care with propofol and fentanyl. Intraoperative hemodynamic variables were recorded and postoperative pain scores were assessed with verbal numerical rating scale (VNRS) 1 min, 15 min, 30 min, 1 h, and 4 h after recovery of consciousness. The primary outcome of our study was total amount of intraoperative and postoperative analgesics administered.

**Results:**

Postoperative serum magnesium concentrations in Group C were significantly decreased than preoperative levels (0.86 ± 0.06 to 0.80 ± 0.08 mmol/L, *P* = 0.001) while there was no statistical change in Group M (0.86 ± 0.07 to 0.89 ± 0.07 mmol/L, *P* = 0.129). Bradycardia did not occur in either group and the incidence of hypotension was comparable between the two groups. Total dose of fentanyl given to patients in Group M was less than the one administered to Group C [100 (75–150) vs 145 (75–175) μg, median (range); *P* < 0.001]. In addition, patients receiving magnesium displayed lower VNRS scores at 15 min, 30 min, 1 h, and 4 h postoperatively.

**Conclusions:**

In hysteroscopy, adjuvant magnesium administration is beneficial to reduce intraoperative fentanyl requirement and postoperative pain without cardiovascular side effects. Our study indicates that if surgical patients have risk factors for hypomagnesemia, assessing and correcting magnesium level will be necessary.

**Trial registration:**

ChiCTR1900024596. date of registration: July 18th 2019.

## Background

Hysteroscopy is currently one of the most common procedures for patients with cervical or endometrial disorders [1]. Although the development of new techniques and equipment has made hysteroscopy a minimally invasive procedure, it’s still believed to be a painful experience which needs effective analgesia to achieve maximum patient comfort and cooperation [2, 3]. Fentanyl is generally the preferred agent administered as analgesics during hysteroscopy because of its low price and powerful analgesic effect. However, serious side effects such as respiratory depressant and postoperative nausea and vomiting (PONV) restrict its dosage in clinical practice [4].

Multimodal analgesia is a strategy that involves the use of two or more analgesic agents and techniques to provide adequate analgesia, and aims to reduce opioid consumption and minimize opioid-related adverse effects [5]. When compared with opioid-free anaesthesia, strong evidence shows that opioid-inclusive anaesthesia does not reduce postoperative pain, but is associated with more PONV [6]. As the fourth most plentiful cation in the body, magnesium (Mg) acts as a non-competitive N-methyl-D-aspartate (NMDA) receptor antagonist and calcium channel blocker. It has antinociceptive stimulus property [7]. Hypomagnesemia is a common entity occurring in up to 12% of hospitalized patients [8] and has been reported in many kinds of surgeries such as thyroidectomy, cardiac surgery, and kidney transplantation. A recent study indicated that serum magnesium level was also significantly decreased after hysteroscopy [9] and magnesium deficiency produces hyperalgesia that can be reversed by NMDA antagonists [10]. In consequence, magnesium administration may be beneficial to patients undergoing hysteroscopy.

We hypothesize that intravenous magnesium sulphate as an adjuvant drug to fentanyl analgesia during hysteroscopy as monitored by Bispectral Index Scale (BIS) could reduce intraoperative and postoperative analgesics requirements and their related side effects.

## Methods

### Ethics and registration

This randomized controlled study adheres to CONSORT guideline. The study was approved by the Ethics Committee of Affiliated Hospital of North Sichuan Medical College [2019ER(R) 074–01] and was registered at the Chinese Clinical Trials Registry (ChiCTR1900024596). Written informed consent was obtained from each patient.

### Patient inclusion and exclusion criteria

Inclusion criteria were patients aged 18–55 years old, with American Society of Anesthesiologists (ASA) physical status I or II, scheduled for hysteroscopy between July 2019 and October 2019 in Affiliated Hospital of North Sichuan Medical College. Exclusion criteria were patients with cardiovascular disease (ejection fraction < 40%, atrioventricular conductance disturbance, hypertension, coronary heart disease, or cerebrovascular disease), liver dysfunction (transaminases above the normal level), renal failure (creatine > 150 μmol/L), preoperative opioids use, neurological disorder, diabetes, body mass index > 30 kg/m^2^, history of neuromuscular disease, history of chronic pain, drugs or alcohol abuse. We also excluded patients when they face serious intraoperative hypoxemia and need endotracheal intubation.

### Randomization and blinding

Patients were randomly assigned into the control group (Group C, *n* = 35) and the magnesium group (Group M, *n* = 35) by computer-generated randomization Web-based, random number generator (available at http://www.random.org). Patients in the Group M received IV magnesium sulfate (Brilliant Pharmaceutical Co., Ltd.) 50 mg/kg in 100 ml of isotonic saline over 15 min before anesthesia induction and then 15 mg/kg per hour by continuous IV infusion until the end of the procedure, whereas patients in the Group C received an equal volume of isotonic saline as a placebo. An anesthetic technician who did not participate in the study was provided with group assignment and prepared the Infusions in pharmacy. Anesthesia provider, patients, and all investigators were blinded to group assignment until completion of the study.

### Anesthesia

On arrival in the operating room, ECG, noninvasive blood pressure (NIBP), and pulse oximetry (SpO_2_) monitoring were commenced. Electrodes were placed on the forehead to monitor bispectral index (BIS). After a 20-G intravenous cannula was inserted, 100 ml of study medicine was started to infusion. Four minutes before the start of procedure, all patients received 1.5 μg/kg bolus doses of fentanyl (Yichang Humanwell Pharmaceutical Co., Ltd.). Sedation was initiated with propofol (Corden Pharma Latina S.p.A) 1.5 mg/kg and then maintained at a rate of 4–12 mg/kg per hour. The speed of propofol infusion was adjusted to maintain a BIS value of 50 to 60. Inadequate analgesia was defined as body movement or an increase in mean blood pressure (MBP) or heart rate (HR) by more than 15% of baseline [4]. A 0.5 μg/kg bolus dose of fentanyl was administered if signs of inadequate analgesia occurred with a BIS value in the recommended range contemporarily. When inadequate analgesia occurred and BIS value simultaneously increased upon 60 or even 70, the speed of propofol infusion was enhanced and a 0.5 μg/kg bolus dose of fentanyl was administered. The infusions of propofol and study medicine were ceased when gynecologists pronounced the completion of the procedure.

During the procedures, all patients were allowed to breathe spontaneously with oxygen 2 L/min via face mask. When SpO_2_ < 95% were observed, patients were managed by jaw thrust and when SpO_2_ < 90% by assisted ventilation. At the same time, ephedrine or atropine was administered if hypotension (SBP ≤ 90 mmHg) or bradycardia (HR ≤ 45bmp) was observed.

### Data collection

After the procedures, patients were transferred to the postanesthesia care unit (PACU) if modified Aldrete score ≥ 9 [11]. Respiratory depression (defined as SpO_2_ less than 95 and 90%), time for recovery of consciousness (time between disconnection of propofol infusion and ability for the patient to provide her name) were recorded. Postoperative pain score was assessed with verbal numerical rating scale (VNRS; 0 = no pain; 4–6 = moderate pain; 10 = worst pain). The VNRS scores were recorded 1 min, 15 min, 30 min, 1 h, and 4 h after recovery of consciousness. If VNRS scores ≥4, bolus doses of dezocine (Yangtze River Pharmaceutical Co., Ltd.) 10 mg used for rescue analgesics were administered intravenously. Patients with PONV were treated with intravenous ondansetron (Qilu Pharmaceutical Co., Ltd.) 4 mg. Gynecologists and Patients’ global satisfaction levels regarding fluency of procedures or comfort level were assessed immediately and 4 h after procedures respectively using a satisfaction scale (0 = complete dissatisfaction; 10 = best satisfaction). Serum magnesium concentrations were collected one day before and one day after the procedure. In addition, PONV and other adverse effects were also recorded during the study period.

### Outcomes

The primary outcome of our study was total amount of intraoperative and postoperative analgesics administered. The following data were collected as secondary outcomes of interest: serum magnesium concentrations, duration of procedure, variations of HR and MBP during procedures, respiratory depression, time for recovery of consciousness, PONV, satisfaction score from gynecologists and patients, VNRS scores after procedures.

### Sample size and statistical analysis

The sample size of this study was based on the total dose of fentanyl requirement. Sample size calculations based on 10 subjects per group were required to achieve a power of 90% with a type 1 error of 0.05. Preliminary data revealed that a total sample size of 62 was required (31 per group) to detect 0.5 μg/kg reduction in fentanyl requirement [12]. In consideration of possible dropout, we enrolled 35 subjects per group.

Data were analyzed using SPSS for Windows version 19.0 (SPSS Inc., Chicago, IL, USA). Normality assessment of distribution was performed with Kolmogorov-Smirnov. Data were set out in the form of mean ± standard deviation, median (range), or the number of patients (proportion). The Student’s t-test was employed in the analysis of the parametric data. Nonparametric data were analyzed by using the Mann-Whitney U test. Categorical data were analyzed using Fisher’s exact test or the chi-square test, if appropriate. Two-way repeated measures ANOVA was used to compare HR and MBP at each point of time. A *P*-value of less than 0.05 was accepted as statistically significant.

## Results

**Fig. 1:**
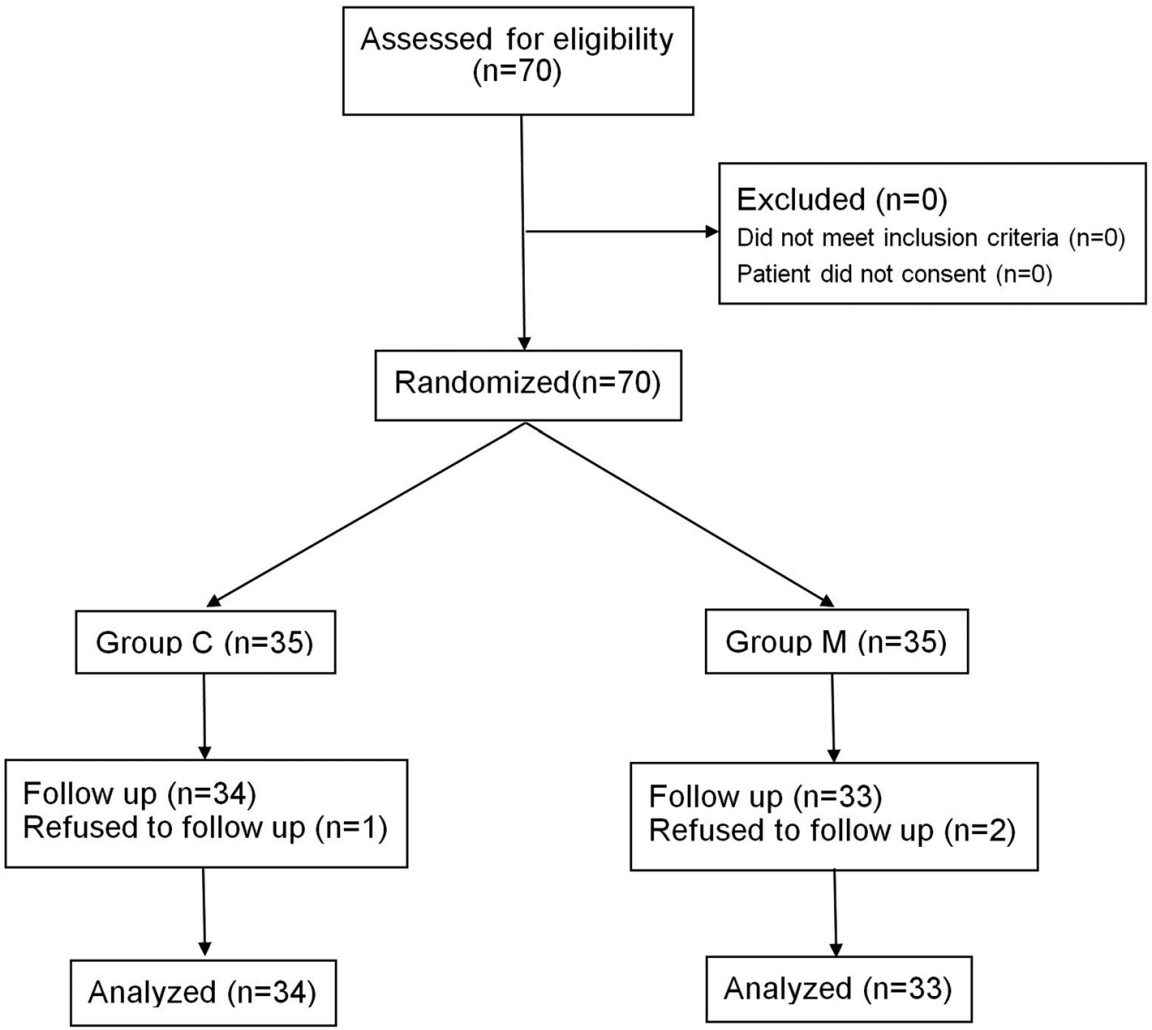
Flow diagram representing patient enrollment, group assignment, and analysis. A total of 70 patients participated in our study without exclusion. They were randomly divided into two groups: the control group (Group C, n = 35) and the magnesium group (Group M, *n* = 35). One patient in Group C and two patients in Group M were lost to follow up, thus 34 patients in Group C and 33 patients in Group M were analyzed

Flow diagram of the study was presented in Fig. 1**:** A total of 70 patients participated in our study without exclusion. They were randomly divided into two groups: the control group (Group C, *n* = 35) and the magnesium group (Group M, n = 35). One patient in Group C and two patients in Group M were lost to follow up, thus 34 patients in Group C and 33 patients in Group M were analyzed. The patients’ demographic characteristics and satisfaction scores are described in Table 1. Age, height, weight, BMI, and ASA physical status were statistically similar between the two groups. There were no significant differences in the duration of procedure and recovery of consciousness. Gynecologists showed higher satisfaction scores in Group M (*P* = 0.026) while patients displayed similar satisfaction scores between two groups (*P* = 0.057).

**Table 1:** Demographic characteristics and satisfaction scores between two groups

	Group C (*n* = 34)	Group M (*n* = 33)	*p* value
Age (years)	37.0 ± 8.8	37.3 ± 8.9	0.900
Weight (kg)	52.5 (44–70)	53.0 (47–65)	0.782
Height (cm)	157.5 ± 4.3	158.1 ± 4.4	0.575
BMI(Kg/m^2^)	22.2 ± 2.5	21.9 ± 2.1	0.618
ASA Physical status (I/II) (n)	13/21	14/19	0.727
Duration of procedure (min)	24.7 ± 12.0	20.4 ± 9.6	0.116
Recovery of consciousness (min)	4 (3–6)	4 (3–5)	0.530
Satisfaction score from gynecologists	8 (7–10)	9 (8–10) ^a^	0.026
Satisfaction score from patients	9 (8–10)	10 (8–10)	0.057

Values are presented as mean ± standard deviation or median (range)

*Group C* Control group, *Group M* Magnesium group

*ASA* American Society of Anesthesiologists

^a^ The difference was significant at 0.05 level

Normal range of serum magnesium level in our institution is 0.75–1.02 mmol/L. Preoperative serum magnesium concentrations were similar between the two groups (0.86 ± 0.06 vs 0.86 ± 0.07 mmol/L in Group C and Group M, respectively). Postoperative serum magnesium concentrations in Group C were significantly declined than preoperative levels (0.86 ± 0.06 to 0.80 ± 0.08 mmol/L, *P* = 0.001) while there was no statistical change in Group M (0.86 ± 0.07 to 0.89 ± 0.07 mmol/L, *P* = 0.129).

The total dose and number of times fentanyl given to patients in Group M was less than these administered to Group C [100 (75–150) vs 145 (75–175) μg, median (range); *P* < 0.001], [2 (1–4) vs 3 (1–5), median (range); *P* < 0.001], meanwhile, propofol consumption was similar between the two groups (*P* = 0.157). Thus, IV magnesium sulphate allowed a 31% reduction in the total dose of fentanyl used during the procedure (Table 2). There was no statistically significant difference for patients who needed rescue analgesic between the two groups [14 vs 6 subjects in Group C and Group M, RR = 0.44 (0.19 to 1.01), *P* = 0.052, NNT 4.349]. All patients’ postoperative pain can be well controlled when they received rescue analgesic for only one time. VNRS scores at 1 min after recovery of consciousness were statistically similar between the two groups but were statistically lower in the Group M at 15 min, 30 min, 1 h, and 4 h postoperatively than in Group C (*P* < 0.05, Table 3). In this study, there was no patient who experienced a VNRS score ≥ 7.

**Table 2:** Anesthetic requirements and frequencies of perioperative adverse events

	Group C (*n* = 34)	Group M (*n* = 33)	*p* value
Propofol (mg)	31.2 ± 10.8	28.0 ± 7.0	0.157
Fentanyl (μg)	145(75–175)	100(75–150) ^a^	< 0.001
Total number of times fentanyl needs	3(1–5)	2(1–4) ^a^	< 0.001
Need for rescue analgesics	14 (41%)	6 (18%)	0.052
Hypertension (SBP > 150 mmHg)	0 (0%)	0 (0%)	
Hypotension (SBP < 90 mmHg)	7 (21%)	5 (15%)	0.562
Tachycardia (HR > 110 bpm)	2 (6%)	0 (0%)	0.157
Bradycardia (HR < 50 bpm)	0 (0%)	0 (0%)	
Respiratory depression			
SpO_2_ < 95%	18 (53%)	12 (36%)	0.172
SpO_2_ < 90%	12 (35%)	7 (21%)	0.201
PONV	4 (12%)	3 (9%)	0.721

Values are presented as mean ± standard deviation, number (proportion) or median (range)

*Group C* Control group, *Group M* Magnesium group

*PONV* Postoperative nausea and vomiting

^a^ The difference was significant at 0.05 level

**Table 3:** Postoperative pain profiles during 4 h

	Group C (*n* = 34)	Group M (*n* = 33)	*p* value
VNRS scores			
1 min	2 (0–4)	1 (0–4)	0.074
15 min	3 (1–6)	2 (1–5) ^a^	0.001
30 min	3 (2–6)	2 (1–6) ^a^	< 0.001
1 h	2 (1–5)	1 (0–5) ^a^	0.001
4 h	2 (1–4)	1 (0–4) ^a^	0.003
VNRS ≥4	14 (41%)	6 (18%)	0.052

Values are presented as median (range) or number (proportion)

*Group C* Control group, *Group M* Magnesium group

*VNRS* Verbal numerical rating scale

^a^ The difference was significant at 0.05 level

**Fig. 2:**
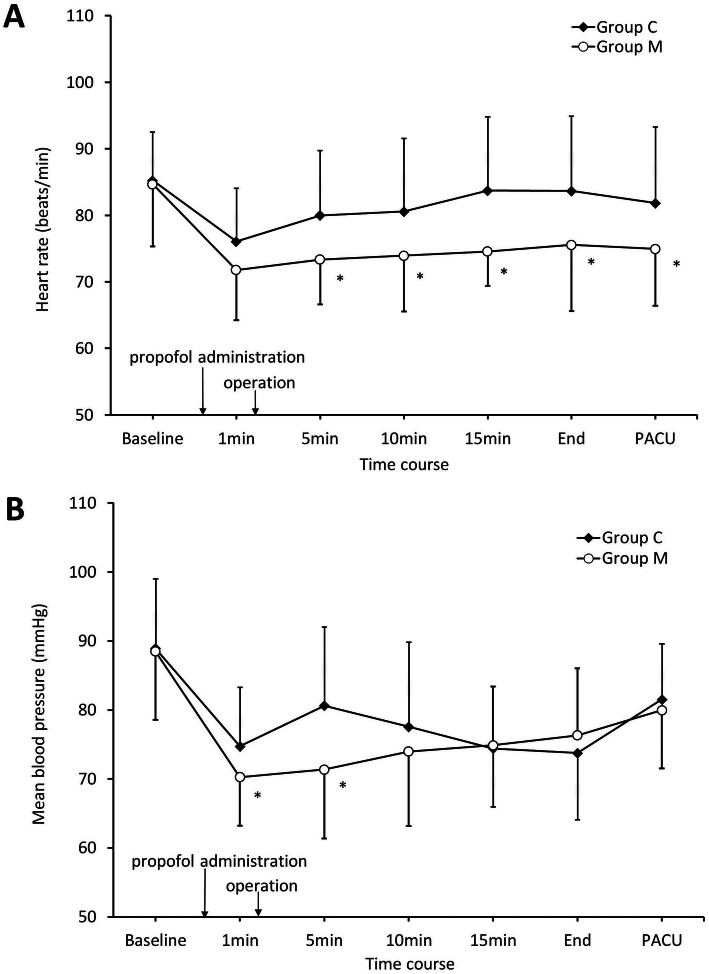
Hemodynamic variables during the procedure. **a** Heart rate in the control group (Group C, *n* = 34) and the magnesium group (Group M, *n* = 33) at each point of time. **b** Mean blood pressure in the control group (Group C, *n* = 34) and the magnesium group (Group M, *n* = 33) at each point of time

Hemodynamic variables during the procedure at each point of time are shown in Fig. 2. A similar trend of heart rate was observed in Group C and Group M, but it was significantly lower in Group M at 5 min, 10 min, 15 min after propofol administration, the end of the procedure, and arrive in PACU. (*P* < 0.05, Fig. 2a). Mean blood pressures at 1 min and 5 min after propofol administration were significantly lower in Group M (*P* < 0.05, Fig. 2b). Hypertension or bradycardia did not occur in either group. Incidence of hypotension was comparable between the two groups and patients were treated with ephedrine when hypotension was observed. However, there was no case of tachycardia in Group M, while two cases were observed in Group C (Table 2). The numbers of patients who experienced oxygen desaturation below 95% (18 vs 12 subjects in Group C and Group M, respectively) or below 90% (12 vs 7 subjects in Group C and Group M, respectively) were statistically insignificant between the two groups **(**Table 2**)**. No patient experienced a serious adverse event related to the infusion of magnesium sulphate.

## Discussion

In the present study, we evaluated the antinociceptive effects of intravenous magnesium sulphate by reducing perioperative analgesics requirements during hysteroscopy in patients under monitored anesthesia care. We demonstrate, in hysteroscopy, that adding intravenous magnesium sulphate to propofol-fentanyl anesthesia results in a reduction in intraoperative fentanyl needs. Patients receiving magnesium displayed slower heart rate and less postoperative pain.

Propofol is an intravenous sedative drug and exerts its effects through potentiation of the inhibitory neurotransmitter, γ-aminobutyric acid (GABA). It has gained widespread use due to its favorable drug effect profile such as rapid and smooth induction with nearly no excitation phenomena and fast terminal half-life time [13]. Fentanyl is an agonist of the μ-opioid receptor which is known to be 100 times more potent than morphine. Analgesic effect occurs as soon as 1 to 2 min and lasts 2 to 4 h [14]. Propofol and fentanyl is metabolized mainly via the liver and excreted in the urine.

Nowadays, hysteroscopic surgeries are frequently performed in ambulatory surgery settings, which benefit the patients for shorter hospital stays and reduction of costs [15]. This procedure has been considered a less invasive treatment, combine short operative time with early discharge, postoperative analgesia is always underestimated and ignored. However, severe pain is caused by uterine cervical dilatation and intrauterine tissue extraction, thus effective pain management is the key point for patients’ comfort and satisfaction. While opioids are the most effective antinociceptive agents, they have undesirable side effects, including respiratory depression, nausea, vomiting, urinary retention, constipation, ileus, and pruritus. Another problem is opioid addiction, a 4.8–6.5% incidence of persistent opioid use after surgery in older children and adults in the United States [16]. With this in mind, opioid-free anesthesia (OFA) was introduced to avoid current crisis. This can be achieved with alpha-2-agonists, ketamine, lidocaine, nonsteroidal anti-inflammatory drugs (NSAIDs) and magnesium, each working on a different target and therefore described as multitarget anesthesia [17]. In hysteroscopy, non-opioid analgesics such as NSAIDs and dexmedetomidine had been evaluated. Although both of these drugs could reduce the pain after hysteroscopy, NSAIDs fail to eliminate the discomfort occurring during the procedure [18] and dexmedetomidine may cause prolonged hypotension and bradycardia [19].

A case report indicated that there is a close connection between hypomagnesaemia and pain. Séamus et al. [20] reported two patients with hypomagnesaemia suffer from severe cancer pain. Their pain was well controlled after treating with intravenous magnesium. During hysteroscopy, distending media is essential to allow for optimal uterine visualization. Nevertheless, excess absorption of large volumes of electrolyte-free, low-viscosity fluid can result in volume overload with hyponatremia and water intoxication [21]. In our study, patients’ postoperative serum magnesium level was consistent with recent research [9] which significantly decreased. The use of diuretics is advocated to treat volume overload in hysteroscopy [21], but diuretics can reduce renal magnesium reabsorption. In the meantime, perioperative inadequate dietary intake of magnesium makes patients undergoing hysteroscopy more susceptible to hypomagnesaemia.

Magnesium has antinociceptive effect in animal and human models of pain [22]. As a matter of fact, noxious stimuli activate the release of glutamate in the dorsal horn, which then activates the NMDA receptors, causing intracellular calcium influx, neuronal excitation, and central sensitization and hyperalgesia [23]. Therefore, NMDA receptor antagonists play an important role in perioperative pain control. Furthermore, compared with acute cutaneous pain sensation, NMDA receptor antagonists provide better pain control for acute visceral pain [24].

Less opioid consumption and better analgesia were observed when patients’ magnesium deficiency was corrected. These observations support both the opioid-sparing effect and co-analgesic properties of magnesium. There are two major mechanisms by which hypomagnesemia can be induced: gastrointestinal or renal losses [8]. As diet is the only source of magnesium, the most common cause of hypomagnesemia in surgical patients is prolonged NPO. Other risk factors include diarrhea, alcoholism, acute pancreatitis, uncontrolled diabetes mellitus, and medication such as a proton pump inhibitor and diuretics [25]. Our results indicate that if patients have these risk factors with complex pain, assessing and correcting magnesium level will be necessary. There is a declining trend for the risk of oxygen desaturation and PONV in the magnesium group, although it did not reach statistically significant. This probably due to short operating time and propofol’s antiemetic effect. Moreover, the recovery of consciousness was not delayed in Group M, while Altan et al. [26] reported that magnesium sulphate caused a delay in recovery for patients undergoing spinal surgery. Magnesium sulphate is known to prolong and potentiate neuromuscular block by non-depolarizing neuromuscular blocking agents [27]. Patients in our study did not receive muscle relaxant and they keep breathe spontaneously. Different surgical model may explain the diverse results on the time of recovery of consciousness between the present study and the result of Altan et al.

Intravenous administration of magnesium generally is associated with minor side effects. Common magnesium-related side effects include flushing, dizziness, and cardiovascular events. Nevertheless, a meta-analysis indicated that magnesium did not have a statistically significant effect on the incidence of dizziness, hypotension, or bradycardia [28]. On the contrary, it was beneficial to reduce postoperative shivering. Hypomagnesaemia can produce numerous symptoms such as pain, weakness, tetany, hallucinations, and arrhythmias [8, 20]. A stable serum magnesium concentration might be helpful for patients’ comfort and postoperative recovery. Jee et al. [29] found that magnesium administration can reduce the release of catecholamine and vasopressin during laparoscopic cholecystectomy. Its antinociceptive effect and direct vasodilatory effect through a calcium channel blockade might explain the lower HR and MBP in Group M. Although there was no significant difference of hypotension between the two groups in our study, relatively lower MBP might be helpful to reduce intraoperative bleeding and stress response. Even though we did not record the specific reasons for adding bolus doses of fentanyl in our study, less fentanyl consumption can reflect fewer times of body movement. These advantages of magnesium sulphate may create good conditions for operation, shorten the duration of procedure, and eventually improve the satisfaction of gynecologists.

Some limitations of the present study should be noted. First, it was a single-center study, and the relatively small number of patients limited the ability to detect statistically significant differences in adverse events of fentanyl between two groups. Second, we only applied magnesium to propofol-fentanyl anesthesia in hysteroscopy. The combination of magnesium with some other medicine in different targets such as lidocaine, ketamine, and dexmedetomidine may be more effective to reduce opioids consumption, even achieve opioid-free anesthesia. Last, we didn’t record magnesium level when we assess postoperative pain scores, so it’s difficult to draw an accurate conclusion on the relationship between magnesium and pain. In further research, magnesium level and pain scores should be assessed dynamically and simultaneously.

## Conclusion

In hysteroscopy, adjuvant magnesium administration is beneficial to reduce intraoperative fentanyl requirement and postoperative pain without cardiovascular side effects. Our study indicates that if surgical patients have risk factors for hypomagnesemia, assessing and correcting magnesium level will be necessary.

## Data Availability

The datasets used during the current study are available from the corresponding author on reasonable request.

## Abbreviations

PONV: Postoperative nausea and vomiting
BIS: Bispectral Index Scale
ASA: American Society of Anesthesiologists
NIBP: Noninvasive blood pressure
SpO2: Pulse oximetry
MBP: Mean blood pressure
HR: Heart rate
PACU: Post anesthesia care unit
VNRS: Verbal numerical rating scale
OFA: Opioid-free anesthesia
